# Early Transcriptional Responses of Bovine Chorioallantoic Membrane Explants to Wild Type, Δ*virB2* or Δ*btpB Brucella abortus* Infection

**DOI:** 10.1371/journal.pone.0108606

**Published:** 2014-09-26

**Authors:** Juliana P. S. Mol, Erica A. Costa, Alex F. Carvalho, Yao-Hui Sun, Reneé M. Tsolis, Tatiane A. Paixão, Renato L. Santos

**Affiliations:** 1 Departamento de Clínica e Cirurgia Veterinárias, Escola de Veterinária, Universidade Federal de Minas Gerais, Belo Horizonte, MG, Brazil; 2 Departamento de Medicina Veterinária Preventiva, Escola de Veterinária, Universidade Federal de Minas Gerais, Belo Horizonte, MG, Brazil; 3 Department of Medical Microbiology and Immunology, School of Medicine, University of California Davis, Davis, California, United States of America; 4 Departamento de Patologia Geral, Instituto de Ciências Biológicas, Universidade Federal de Minas Gerais, Belo Horizonte, MG, Brazil; Federal University of Pelotas, Brazil

## Abstract

The pathogenesis of the *Brucella*-induced inflammatory response in the bovine placenta is not completely understood. In this study we evaluated the role of the *B. abortus* Type IV secretion system and the anti-inflammatory factor BtpB in early interactions with bovine placental tissues. Transcription profiles of chorioallantoic membrane (CAM) explants inoculated with wild type (strain 2308), Δ*virB2* or Δ*btpB Brucella abortus* were compared by microarray analysis at 4 hours post infection. Transcripts with significant variation (>2 fold change; P<0.05) were functionally classified, and transcripts related to defense and inflammation were assessed by quantitative real time RT-PCR. Infection with wild type *B. abortus* resulted in slightly more genes with decreased than increased transcription levels. Conversely, infection of trophoblastic cells with the Δ*virB2* or the Δ*btpB* mutant strains, that lack a functional T4SS or that has impaired inhibition of TLR signaling, respectively, induced more upregulated than downregulated genes. Wild type *Brucella abortus* impaired transcription of host genes related to immune response when compared to Δ*virB* and Δ*btpB* mutants. Our findings suggest that proinflammatory genes are negatively modulated in bovine trophoblastic cells at early stages of infection. The *virB* operon and *btpB* are directly or indirectly related to modulation of these host genes. These results shed light on the early interactions between *B. abortus* and placental tissue that ultimately culminate in inflammatory pathology and abortion.

## Introduction

Brucellosis is an important zoonotic disease with worldwide distribution, caused by bacteria of the genus *Brucella*. It causes significant economic losses due to abortions and culling of infected cattle, whereas in humans it is associated with a febrile illness with variable symptoms, and it may occasionally be fatal [1]–[3].

Most cases of bovine brucellosis are due to *Brucella abortus* infection, which is transmitted by contact with contaminated aborted fetuses, fetal membranes, and uterine secretions after abortion or during the postpartum period [4], [5]. Aborted fetuses resulting from *B. abortus* infection often exhibit signs of fibrinous pleuritis, which may be associated with suppurative bronchopneumonia and fibrinous pericarditis [6], [7]. During pregnancy, after the initial infection of the erythrophagocytic trophoblasts located at the base of the chorionic villi, the bacteria spread throughout the placenta following a periplacentomal pattern, infecting trophoblastic cells of the intercotyledonary region, mostly at the end of gestation (180 to 240 days) [8]–[11]. Thus, *B. abortus* triggers an intense acute inflammatory response in the placenta, which is associated with abortion [7]. While this inflammatory pathology is well-described, very little is known about the initial interactions between *B. abortus* and placental cells that ultimately result in placentitis and abortion, two processes that are key components of disease transmission. Because of the difficulty of studying these early interactions in pregnant animals, *ex vivo* infection of cultured chorioallantoic membrane (CAM) explants, which results in localization of *B. abortus* to trophoblasts, has been used to study the initial phases of placental infection [12], [13].

Virulence factors of *B. abortus* have been studied in the context of persistent infection of the mononuclear phagocyte system, however few studies have been performed in the context of placental infection in the natural host. The type IV secretion system (T4SS) is considered to be a key virulence factor of *Brucella* spp., and it is responsible for secretion of effector proteins across the bacterial cell envelope [14]–[16]. The T4SS has been shown to be involved in abortion in goats [17], raising the question of its contribution to early interactions with the placenta.

A second set of virulence factors, shown to be involved in immune evasion, are the TIR domain proteins, BtpA and BtpB [18]–[20]. BtpA is present in *B. abortus*, *B. melitensis*, and *B. suis* ATCC 23445 (biovar 2), but it is absent in *B. suis* 1330 (biovar 1). BtpA binds directly to MyD88, preventing signaling via TLR2 and TLR4, impairing innate immune response and inhibiting maturation of *in vitro* infected dendritic cells by blocking the TLR2 signaling pathway [18], [20]. BtpB is present in all species of *Brucella*. It inhibits TLR signaling, preventing activation of dentritic cells, and together with BtpA can modulate the host inflammatory responses during *Brucella* sp. infection [19]. Interestingly, translocation of BtpB into host macrophages was shown to depend on the VirB T4SS [19].

In this study, the CAM explant model was used as an *ex vivo* model to study the pathogenesis of *Brucella* infection during the initial phase of the bacteria-host interaction [12]. Carvalho Neta et al [13], using this model, demonstrated that *B. abortus* modulates the innate immune response by trophoblastic cells by inhibiting the transcription of proinflammatory mediators at early stages of infection. The aim of this study was to interrogate the role of the VirB T4SS and BtpB in early suppression of inflammatory responses in the placenta.

## Materials and Methods

### Bacterial strains

The inocula were prepared by growth for 12–15 hours under agitation at 37°C of the three bacterial strains: *B abortus* 2308 was cultivated in *Brucella* broth (Difco, Lawrence, KS, USA) and the Δ*virB2 B. abortus* and Δ*btpB B. abortus* strains were cultivated in Tryptic Soy Broth (Difco) supplemented with kanamycin (100 µg/µL). After incubation, the optical density of bacterial suspensions was determined by spectrophotometry (OD_600_) and adjusted to 1.0×10^8^ CFU/mL. To confirm the concentration of bacteria, the inocula were serially diluted in PBS (pH 7.4), and 100 µL of each dilution were plated on Tryptic Soy Agar (Difco), in duplicate. After 48 h of incubation at 37°C with 5% CO_2_, colonies counted and the number of colony forming units (CFU) was obtained by the average of duplicates. The handling of agent and infected material was performed under biosafety level 3 containment.

### Generation of mutant strains

The Δ*virB2 B. abortus* strain used in this study was obtained by allelic exchange of the *virB2* gene (BAB2_0067), inserting a kanamycin cassette. The plasmid used to construct the mutant strains was the pAV2.2, a kanamycin resistant vector, which has been described previously [21]. The Δ*btpB B. abortus* strain was obtained by allelic exchange of the *btpB* gene (BAB1_0279) for a kanamycin cassette. The plasmid used for *btpB* mutagenesis was generated in this study. For this purpose a plasmid called pUKD/*btpB* was made by using a previously described three-step cloning strategy [22]. Briefly, a fragment of the 5′ region of BAB1_0279 with engineered *Sma*I site was amplified from *B. abortus* genomic DNA using primers BMEI1674UP-F (TGAATGTGGCAAGCCCTCGAC) and BMEI1674UP-R (ACCCGGGCTTGTTTCTCTTTAGAC). A fragment of the 3′ region of BAB1_0279 with engineered *Sma*I and *Pst*I sites was amplified using primers BMEI1674DN-F (ACCCGGGCAGATGCAAATATGGCCGTAAG) and BMEI1674DN-R (TCTGCAGCCGGAGGAATGGCATCAC). Both amplicons were TOPO cloned into pCR2.1 to yield plasmids pUP/*btpB* and pDN/*btpB* respectively. The orientation of the inserted 5′ fragment of BAB1_0279 was determined by PCR and restriction analysis to make sure the unique *Sma*I site was next to the T7 promoter in pCR2.1 vector. The 3′ fragment of BAB1_0279 was then excised by *Sma*I/*Pst*I double digestion and cloned into the same sites of pUP/*btpB* to generate the pUD/*btpB*. The resulting plasmid was selected for ampicillin resistance as double digestion of *Sma*I/*Pst*I truncates the original kanamycin resistance gene in pCR2.1. In the third cloning step, a 1.3-kb *Sma*I fragment of pUC4-KIXX (Pharmacia) containing the Tn*5* kanamycin resistance gene was cloned into the *Sma*I site of pUD/*btpB* to give rise topUKD/*btpB*, which was selected for both kanamycin and ampicillin resistance. These plasmids were transformed into electrocompetent *B. abortus* cells by electroporation as previously described [23]. Colonies that were kanamycin resistant and ampicillin sensitive were selected as mutant candidates. Deletion of the *virB2* and *btpB* genes was confirmed by PCR using primers flanking the deleted region.

### Chorioallantoic membrane explant culture and infection

Snapwell plates (Transwell Cell Culture Permeable Supports – Snapwell Inserts - Corning Incorp., NY, EUA) were used for culturing CAM explants [12], [13]. Seven intact pregnant bovine uteruses at the final third of gestation were obtained at local slaughterhouses. Gestational age was estimated by cephalococcygeal length (CR - Crown-rump length) [24]. All fetuses were serologically negative for *Brucella* spp. by using the Acidified Antigen Buffered test with fetal amniotic fluid. Prior to obtaining the CAM, the perimetrium was thoroughly decontaminated with iodinated alcohol, the uterus was then opened and CAM removed and placed in RPMI 1640 sterile medium (Invitrogen, São Paulo, Brazil) containing antibiotics (100 U/mL penicillin and 100 µg/mL streptomycin) for 20 minutes. CAM explants were then washed twice in RPMI (Invitrogen) at 37°C for complete removal of the antibiotic. Sterile rings and detachable supports were positioned over the intercotyledonary portion of the CAM explants. Excess tissue was removed from CAM explants, which were placed in 6 well culture plates (Corning, NY, USA) containing sterile culture medium RPMI 1640 supplemented with 4 mM glutamine, 1 mM pyruvate, 1 mM nonessential amino acids, 2.9 mM sodium bicarbonate and 15% fetal bovine serum (Invitrogen), and incubated at 37°C in a humidified atmosphere with 5% CO_2_. The experimental protocol was approved by the Ethics Committee on Animal Experimentation of UFMG (CETEA – Protocol 183/2010).

The trophoblastic surface of the CAM explants was inoculated with 200 µL of culture medium (RPMI 1640) containing 2.0×10^7^ CFU, which corresponded to a multiplicity of infection of approximately 1000 (MOI 1000∶1) as previously used by Carvalho Neta et al. [13]. CAM explants were inoculated in triplicate with wild type, Δ*virB2* or Δ*btpB* strains of *B. abortus* 2308. Plates were incubated at 37°C with 5% CO_2_ for 4 h, the medium was then replaced with RPMI 1640 medium supplemented with 50 µg/mL gentamicin (Invitrogen, São Paulo, Brazil) to inactivate extracellular bacteria. Plates were maintained at 37°C with an atmosphere containing 5% CO_2_ for 1 h followed by washing three times with PBS (phosphate buffered saline - pH 7.4) to eliminate the antibiotic. Uninfected CAM controls were inoculated with sterile RPMI 1640 medium and kept under the same conditions.

### Determining the number of internalized bacteria

To determine the number of internalized bacteria, three explants inoculated with wild type, Δ*virB2* or Δ*btpB B. abortus* 2308 were incubated for 4 h, followed by 1 h incubation with RPMI 1640 medium supplemented with 50 µg gentamicin/mL (Invitrogen, São Paulo, Brazil) to inactivate extracellular bacteria, washed three times with PBS (pH 7.4), and then lysed with 200 µL of sterile 0.1% Triton X-100 (Roche, Mannheim, Germany). Serial dilutions of the lysates were prepared in PBS (pH 7.4), and 100 µL of each dilution were plated on tryptose agar (Difco) in duplicate. After 48 h of incubation at 37°C with 5% CO_2_, the number of colony forming units (CFU) was counted in each plate.

### RNA extraction and preparation of cDNA

After removal of RPMI culture medium supplemented with gentamicin (n = 4 for microarray analysis; n = 3 for qRT-PCR), TRIzol Reagent (Invitrogen) was added to the trophoblastic surface of the CAM explants of uninfected controls or explants infected with wild type, Δ*virB2* or Δ*btpB B. abortus* 2308, for total RNA extraction, according to the manufacturer's instructions. Purity and concentration of RNA samples were assessed by spectrophotometry, and RNA quality evaluated by agarose/formaldehyde gel electrophoresis. RNA samples were stored at −80°C. Synthesis of cDNA was performed using Superscript III First Strand S (Invitrogen), following the manufacturer's specifications using a RNA concentration of 1,500 ng in a reaction with a final volume of 20 µL, and cDNA was stored at −20°C.

### Microarray analysis

Gene expression profiles were evaluated using the Agilent two color microarray-based gene expression platform according to manufacturer's instructions. Briefly, RNA (500 ng) was amplified and labeled using the two-color Quick Amp labeling kit (Agilent Technologies, CA, USA). Complementary RNA (cRNA) was synthesized from triplicates of CAM explants of four independent experiments. cRNA from uninfected control explants labeled with Cy3 and cRNA from explants infected with either wild type, Δ*virB2* or Δ*btpB B abortus* 2308, labeled with Cy5 were hybridized on the same slide at 65°C, for 17 hours and 10 RPM in a high-density microarray containing 4×44,000 genes representing fully sequenced bovine genome (#G2519F Agilent Technologies, Palo Alto, CA, USA). After hybridization, slides were washed in Gene Expression wash buffers 1 and 2 as per instructions, scanned with an Agilent DNA microarray scanner (Agilent Technologies) and the hybridization signals were extracted using the Agilent Feature Extraction software version 11.0.

### Quantitative real-time PCR (qRT-PCR)

After functional classification using the Funcat (Functional Classification) platform (http://mips.helmholtzmuenchen.de/genre/proj/mfungd/Search/Catalogs/searchCatfirstFun.html) genes of interest, i.e. related to inflammation and immune response that had at least a 2-fold change in transcription levels and that were statistically significant (P<0.05) were selected for confirmation based on qRT-PCR. Levels of transcripts were normalized based on β-actin transcript level. qRT-PCR was performed using the SYBR Green PCR Master Mix (Applied Biosystems, NY, USA) and the StepOnePlus thermal cycler (Applied Biosystems, NY, USA). Primers used in this study are described in Table 1. The data were analyzed using the comparative Cycle threshold (Ct) method [25].

**Table 1:** Primers used in this study.

Gene	Primers (5′-3′)	Product size (nt)
IFN-alpha G	TCAAGCCATCTCTGTGCTCC	72
	ACGGCTGAACCCTCTACACT	
Chemokine (C-X-C motif) ligand 12	GATGCCAAGGTCTTCGTCGT	104
	TCAAAGAATCGGCAAGGGCA	
Interleukin 15	TGGGCTGTATCAGTGCAAGT	148
	ACTTTGCAATTGGGATGAGCA	
Interleukin 1 family, member 6 (epsilon)-like	GCCGGAGCTTTGTCTCTTCT	136
	CCTGCCATTCTGGTCATGGT	
Transmembrane 4 L six family member 19	CCCTGCCGAAGGATGCTTAT	85
	GCAAATCAAGGCTCCAAGCA	
Tumor necrosis factor receptor superfamily, member 9	ACATGGCATCTGTCGACCTT	82
	ACGTCACTTTCCTTCGTCCC	
Apolipoprotein L, 3	CAGAGACACACGAAAGGCG	108
	GGCTGGAAGAAGGTGTCGTT	
Heat shock 70 kDa protein 1- like	AAACTGGATCGAAGGCGGC	53
	GCTGCAGCCATGATTTTCCT	
Platelet/endothelial cell adhesion molecule	GCTGACCCTTCTGCTCTGTT	116
	GTGTCAGGTTCTCCCCGTTT	
Pellino homolog 2	CCCAATAAGGAGCCCGTGAA	136
	TGGGTTTGACTCCGTTAGCC	
β-actina	ACTTGCGCAGAAAACGAGAT	84
	CACCTTCACCGTTCCAGTTT	

### Statistical analysis

Normalization and statistical analysis of the microarray data were performed using the GeneSpring software (Agilent Technologies, CA, USA). Analysis of variance (ANOVA) and the Student's *t*-test were performed with significance level of P<0.05. Analysis of variance (ANOVA) was performed after logarithmic transformation of CFU values and means were compared by the Tukey's Multiple Comparison Test (P<0.05).

### Microarray data accession number

The microarray data set has been submitted to the Gene Expression Omnibus database at NCBI (http://www.ncbi.nlm.nih.gov/geo/) and assigned accession number GSE58216.

## Results

### Internalization of wild type, *ΔvirB2* and *ΔbtpB Brucella abortus* strains by bovine trophoblastic cells

**Figure 1:**
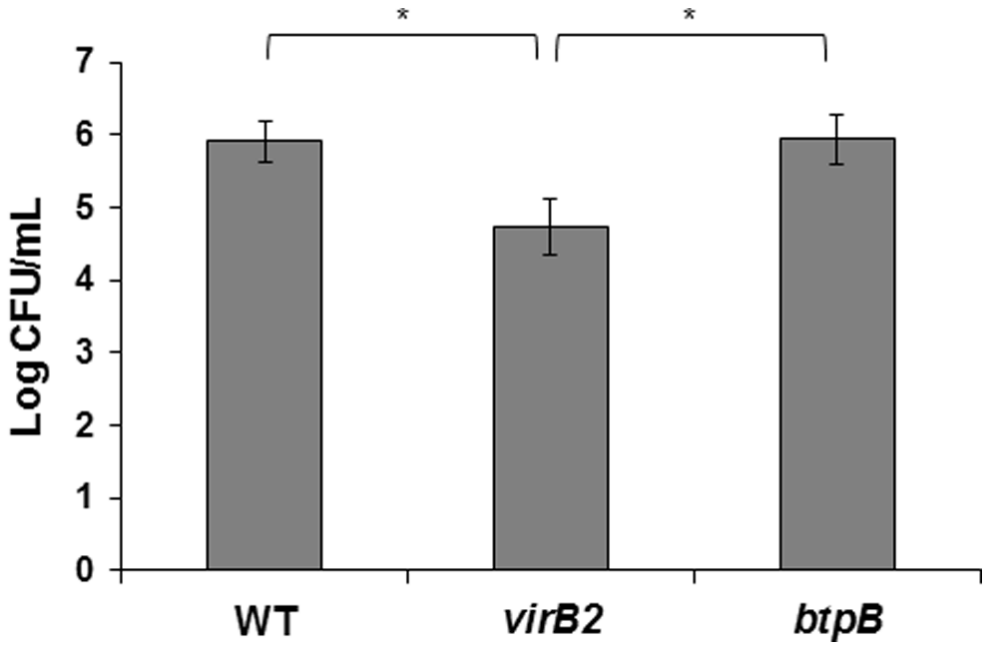
Internalization of wild type, Δ*virB2* or Δ*btpB Brucella abortus* by bovine trophoblastic cells. Chorioallantoic membrane (CAM) explants were inoculated, incubated for 4 h, followed by 1 h incubation with gentamicin, and then lysed for intracellular CFU counting. Data represents the average log of CFU numbers from CAM explants, from three independent experiments performed in triplicates. Data underwent logarithmic transformation followed by analysis of variance (ANOVA) and the Tukey's multiple comparison test with significance level of P<0.05.

In order to evaluate whether the *Brucella* strains used in this study had comparable levels of internalization in trophoblastic cells of bovine CAM explants, CFU numbers at 4 h post inoculation followed by 1 h of incubation with gentamicin. There was no difference between the number of internalized wild type and Δ*btpB B. abortus* 2308. It has been demonstrated that in infected CAM, *B. abortus* is found intracellularly in trophoblasts [13]. In contrast, the number of internalized Δ*virB2* mutant *B. abortus* was significantly lower than the other two strains (Figure 1).

### Transcription profile of bovine trophoblastic cells during infection with wild type, *ΔvirB2* or *ΔbtpB Brucella abortus* 2308 strains

**Figure 2:**
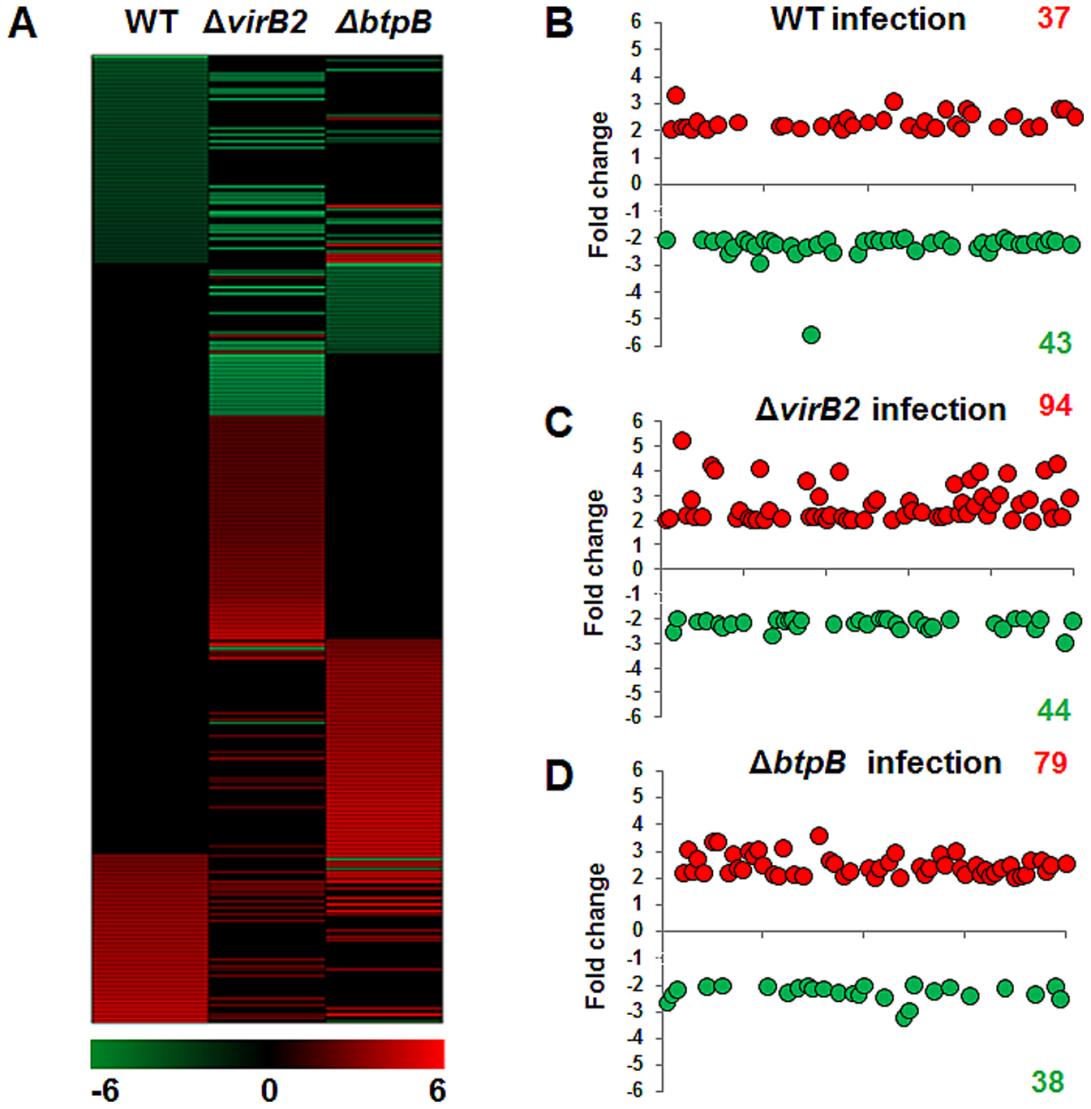
Gene transcription profiling of the host response to *B. abortus* strains at 4 hours after infection of bovine trophoblastic cells. (A) A heat map of gene transcription changes in bovine trophoblastic cells infected with wild type, Δ*virB2* and Δ*btpB B. abortus*– strains, compared to mock-infected controls. (B) Fold changes in gene transcription for genes that were significantly (P<0.05) up or downregulated during wild type, Δ*virB2* and Δ*btpB B. abortus* infection compared to mock-infected controls. These data represent results from pools of total RNA obtained from triplicates of CAM explants obtained from four independent experiments. Increase or decrease in mRNA levels are indicated in red or green, respectively.

**Figure 3:**
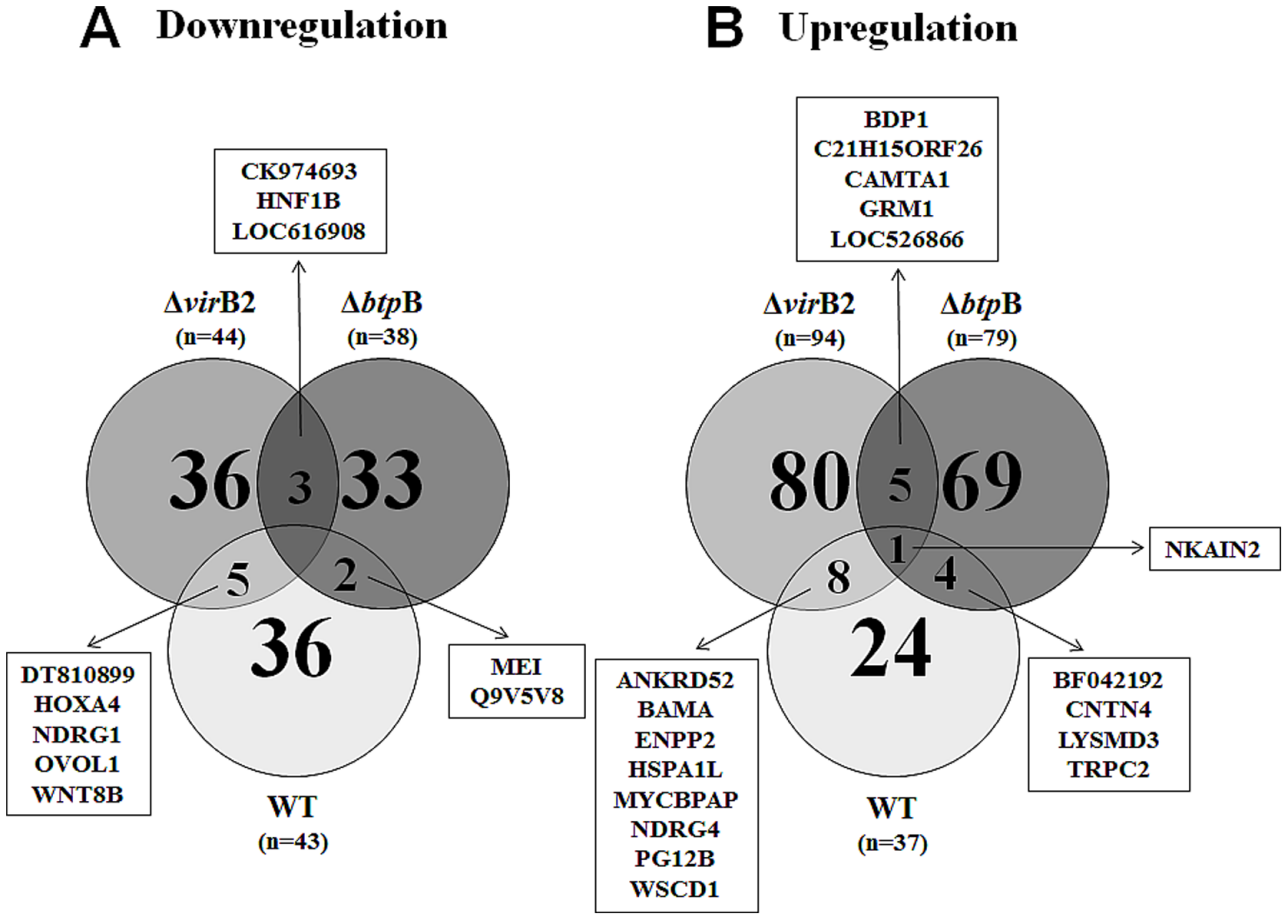
Venn diagram indicating the number of genes with significant changes in mRNA levels assessed by microarray analysis in bovine trophoblastic cells from CAM explants obtained from 4 placentas at the last trimester of pregnancy (n = 4) infected with wild type, Δ*virB2* and Δ*btpB B. abortus* compared to mock-infected controls. Changes in transcription higher than 2-fold and values of P<0.05 were considered significant. (A) Downregulated genes and (B) upregulated genes. Abbreviations: 4105740 BARC 9BOV cDNA clone 9BOV30_O11 5′ (CK974693), HNF1 homeobox B (HNF1B), Hypothetical protein LOC616908 (LOC616908), LB01613.CR_H15 GC_BGC-16 cDNA clone IMAGE:8082593 (DT810899), Homeobox A4 (HOXA4), N-myc downstream regulated 1 (NDRG1), Ovo-like 1 (*Drosophila*) (OVOL1), PREDICTED: wingless-type MMTV integration site family, member 8B (WNT8B), Hypothetical LOC516011 (MEI), Q9V5V8_DROME CG13214-PA, isoform A (Q9V5V8), BDP1 protein Fragment (BDP1), Chromosome 15 open reading frame 26 ortholog (C21H15ORF26), Calmodulin binding transcription activator 1 (CAMTA1), GRM1 protein Fragment (GRM1), hCG1653800-like (LOC526866), Na+/K+ transporting ATPase interacting 2 (NKAIN2), Ankyrin repeat domain 52 (ANKRD52), 001128BAMA005012HT BAMA cDNA (BAMA), Ectonucleotide pyrophosphatase/phosphodiesterase 2 (ENPP2), Heat shock 70 kDa protein 1-like (HSPA1L), Hypothetical LOC505551 (MYCBPAP), NDRG family member 4 (NDRG4), PG12B_HUMAN (Q9BX93) Group XIIB secretory phospholipase A2-like protein precursor [TC318659] (PG12B), WSC domain containing 1 (WSCD1), BP250013A20E1 Soares normalized bovine placenta cDNA (BF042192), Contactin 4, isoform c precursor (CNTN4), LysM, putative peptidoglycan-binding, domain containing 3 (LYSMD3), Transient receptor potential channel 2 (TRPC2).

Considering that *B. abortus* modulates the innate immune response of bovine trophoblastic cells [13], and that TIR domain-containing *Brucella* proteins, such as BtpB have been shown to impair the host innate immune response [19], whereas the *virB*-encoded T4SS is required for *Brucella* survival within host cells [26], a comparison of the transcription profile of bovine trophoblastic cells infected with wild type *B. abortus*, or the isogenic Δ*virB2* or Δ*btpB* strains was performed in this study. A heat map was generated to analyze transcripts with >2 fold change that had statistically significant differences in expression (P<0.05) (Figure 2). Infection of bovine trophoblastic cells with wild type *B. abortus* 2308 resulted in 80 transcripts with differential levels of expression (i.e. at least a 2-fold change). Among those transcripts, 37 were upregulated and 43 were downregulated. In contrast, infection with Δ*virB2* or Δ*btpB B. abortus* mutant strains resulted in a higher number of differentially expressed transcripts (138 and 117, respectively). While the number of downregulated genes was similar between wild type *B. abortus* and the mutant strains, remarkably, we observed an increased number of upregulated genes in CAM explants infected with both the Δ*virB2* and the Δ*btpB* mutants (Figure 2 and 3, Table 2).

**Table 2:** Genes with significant decrease in transcription level in bovine trophoblastic cells (chorioallantoic membrane explants) infected with wild type (strain 2308), Δ*virB2*, or Δ*btpB* in comparison to uninfected controls at 4 hours post infection.

Function* and GenBank identification	Strain**	Fold Change	P value
**Cell biogenesis**			
NOMO3-like protein Fragment [ENSBTAT00000046252]	Δ*btpB*	2.180	0.0420
tubulin polymerization promoting protein [NM_173976]	Δ*btpB*	2.353	0.0004
LMNB2_HUMAN (Q03252) Lamin-B2 [TC345309]	Δ*virB2*	2.338	0.0467
microfibrillar associated protein 5 [NM_174386]	Δ*virB2*	2.651	0.0089
keratin 10 [NM_174377]	WT	2.554	0.0319
**Cell cycle/DNA processing**			
AT rich interactive domain 1A, transcript variant 1 [XM_592084]	Δ*btpB*	2.361	0.0151
hypothetical LOC516011 [XM_594137]	Δ*btpB*	2.031	0.0048
nuclear receptor subfamily 2, group E, member 3 [NM_001167900]	Δ*btpB*	3.237	0.0254
SWI/SNF related, matrix associated [NM_001172224]	Δ*btpB*	2.116	0.0012
centrosomal protein 97 kDa [NM_001192424]	Δ*virB2*	2.201	0.0321
ephrin-A5 [NM_001076432]	Δ*virB2*	2.205	0.0113
hepatocyte nuclear factor 4, alpha [NM_001015557]	Δ*virB2*	2.367	0.0496
misc_RNA (ETV4), miscRNA [ENSBTAT00000010973]	Δ*virB2*	2.057	0.0492
ovo-like 1 [NM_001081521]	Δ*virB2*	2.112	0.0196
polymerase I and transcript release factor [NM_001081731]	Δ*virB2*	2.908	0.0010
zinc finger, ZZ-type with EF-hand domain 1 [XM_864249]	Δ*virB2*	2.435	0.0063
hypothetical LOC516011 [XM_594137]	WT	2.109	0.0476
ovo-like 1 [NM_001081521]	WT	2.082	0.0145
Q6P1W9_HUMAN Histone deacetylase 7 A protein [TC363092]	WT	2.041	0.0057
**Cell fate**			
triple functional domain (PTPRF interacting) [ENSBTAT00000007252]	Δ*virB2*	2.306	0.0159
**Defense and inflammation**			
CD200 molecule [NM_001034620]	Δ*btpB*	2.073	0.0250
platelet/endothelial cell adhesion molecule [NM_174571]	Δ*btpB*	2.240	0.0460
N-myc downstream regulated 1 [NM_001035009]	Δ*virB2*	2.189	0.0192
polymeric immunoglobulin receptor [NM_174143]	Δ*virB2*	2.211	0.0280
chemokine (C-X-C motif) ligand 12 mRNA [NM_001113174]	WT	5.527	0.0436
N-myc downstream regulated 1 [NM_001035009]	WT	2.222	0.0349
pellino homolog 2 [XM_612354]	WT	2.040	0.0366
serine peptidase inhibitor. Kazal type 5 [NM_001102102]	WT	2.118	0.0015
TRAF2 and NCK interacting kinase [ENSBTAT00000015600]	WT	2.211	0.0097
**Signal transduction**			
oculocerebrorenal syndrome of [NM_001102191]	Δ*btpB*	2.956	0.0060
odorant receptor MOR10-like [XM_591864]	Δ*btpB*	4.476	0.0062
neurogranin (protein kinase C substrate. RC3) [NM_001113313]	Δ*virB2*	2.414	0.0222
olfactory receptor Olfr399-like [XM_002695734]	Δ*virB2*	2.214	0.0109
olfactory receptor. family 13. subfamily C. member 3-like [XM_001256440]	Δ*virB2*	2.003	0.0077
wingless-type MMTV integration site family. member 8B [XM_582222]	Δ*virB2*	2.017	0.0492
olfactory receptor Olr136-like [XM_002693219]	WT	2.106	0.0471
olfactory receptor Olr1654-like [XM_001255032]	WT	2.001	0.0205
olfactory receptor Olr1654 [XM_001255418]	WT	2.025	0.0407
olfactory receptor. family 4. subfamily K. member 13-like [XM_001255280]	WT	2.048	0.0460
phosphodiesterase 7A [ENSBTAT00000015427]	WT	2.025	0.0092
wingless-type MMTV integration site family. member 5B [XM_584724]	WT	2.434	0.0412
wingless-type MMTV integration site family. member 8B [XM_582222]	WT	2.283	0.0412
**Transport**			
1254442 MARC 7BOV cDNA 5′ [DN516021]	Δ*btpB*	2.659	0.0189
ELMO/CED-12 domain containing 1 [NM_001078108]	Δ*btpB*	2.135	0.0376
ArfGAP with SH3 domain. ankyrin repeat and PH domain 2 [ENSBTAT00000003007]	Δ*virB2*	2.003	0.0184
sodium channel. voltage-gated. type V. alpha subunit [NM_174458]	Δ*virB2*	2.216	0.0095
syntaxin-1B (Syntaxin-1B2) (Synaptocanalin I) [ENSBTAT00000044107]	Δ*virB2*	2.020	0.0409
discs. large homolog [NM_001191307]	WT	2.132	0.0067
N-ethylmaleimide-sensitive factor attachment protein. beta [NM_001046233]	WT	2.122	0.0228
potassium voltage-gated channel. shaker-related subfamily. beta member 1 [NM_001025336]	WT	2.870	0.0002
RAB6A. member RAS oncogene family (RAB6A). mRNA [NM_001193115]	WT	2.477	0.0284
**Systemic development**			
Ellis van Creveld syndrome 2 [NM_173927]	Δ*btpB*	2.030	0.0344
HNF1 homeobox B [NM_001192855]	Δ*btpB*	2.286	0.0035
HUMHRX {Homo sapiens} (exp = −1; wgp = 0; cg = 0) [TC345413]	Δ*btpB*	2.170	0.0209
myeloid/lymphoid or mixed-lineage leukemia [NM_001192549]	Δ*btpB*	2.464	0.0004
HNF1 homeobox [NM_001192855]	Δ*virB2*	2.426	0.0167
homeobox A4 [NM_001076134]	Δ*virB2*	2.315	8 E-05
mitofusin 2 (MFN2). transcript variant 1 [NM_001190269]	Δ*virB2*	2.371	0.0020
myeloid/lymphoid or mixed-lineage leukemia [NM_001192025]	Δ*virB2*	2.031	0.0124
homeobox A4 [NM_001076134]	WT	2.005	0.0010
**Interaction with environment**			
contactin-4-like [XM_600040]	Δ*btpB*	2.485	0.0004
extracellular matrix protein 2. female organ and adipocyte specific [NM_001034597]	Δ*btpB*	2.150	0.0377
neurocan (NCAN). mRNA [NM_001193082]	Δ*virB2*	2.201	0.0117
Neuronal cell adhesion molecule Fragment [ENSBTAT00000008864]	Δ*virB2*	2.047	0.0035
binder of sperm 1[NM_001001145]	WT	2.185	0.0221
neuroligin 2 [NM_001191242]	WT	2.001	0.0026
SCO-spondin homolog mRNA [NM_174706]	WT	2.143	0.0118
**Metabolism**			
4-hydroxyphenylpyruvate dioxygenase-like [NM_001099371]	Δ*btpB*	2.038	0.0057
acyl-CoA dehydrogenase. long chain (ACADL) [NM_001076936]	Δ*btpB*	2.022	0.0375
inter-alpha (globulin) inhibitor H3 [NM_001101898]	Δ*virB2*	2.112	0.0272
phospholipase A2. group [NM_001193052]	Δ*virB2*	2.127	0.0342
pipecolic acid oxidase [NM_001014878]	Δ*virB2*	2.206	0.0239
steryl-sulfatase-like [XM_001789191]	Δ*virB2*	2.039	0.0008
adenosine deaminase. RNA-specific [ENSBTAT00000009896]	WT	2.342	0.0200
ADP-ribosyltransferase 5 [NM_001076515]	WT	2.204	0.0048
phosphoinositide-3-kinase. catalytic. delta polypeptide [XM_580673]	WT	2.074	0.0283
retinol dehydrogenase 12 (all-trans/9-cis/11-cis) [NM_183363]	WT	2.189	0.0146
**Protein fate**			
leucine rich repeat containing 41 (LRRC41) [NM_001045873]	Δ*btpB*	2.372	0.0161
peptidylprolyl isomerase (cyclophilin)-like 2 [NM_001038081]	Δ*btpB*	2.014	0.0093
ribosomal protein S6 kinase. 90 kDa. polypeptide 4 [NM_001191400]	Δ*btpB*	2.390	0.0029
F-box protein 32 [NM_001046155]	Δ*virB2*	2.341	0.0159
**Protein synthesis**			
eukaryotic elongation factor-2 kinase (EEF2K). mRNA [NM_001192542]	Δ*virB2*	2.056	0.0075
alanyl-tRNA synthetase 2. mitochondrial (putative) [NM_001191211]	WT	2.066	0.0296
**Binding function**			
EH domain binding protein 1-like 1 [NM_001191243]	Δ*btpB*	2.120	0.0095
PR domain containing 13 [NM_001193010]	Δ*btpB*	2.078	0.0181
suppressor of sable-like [XM_582657]	Δ*btpB*	2.582	0.0487
syndecan binding protein (syntenin) 2 [ENSBTAT00000039310]	Δ*virB2*	2.171	0.0358
**Regulation of metabolism**			
cystatin SC [NM_001038122]	Δ*btpB*	2.276	0.0247
Serine protease inhibitor Kazal-type 6 Precursor [ENSBTAT00000047606]	Δ*btpB*	2.212	0.0325
dedicator of cytokinesis 4 [XM_613918]	Δ*virB2*	2.006	0.0180
LB00228.CR_H15 GC_BGC-02 cDNA clone IMAGE:7950401 [DT823428]	WT	2.067	0.0031
**Transcription**			
Q5TAY6_HUMAN (Q5TAY6) Zinc finger protein 31 [TC342188]	Δ*btpB*	2.108	0.0298
zinc finger and BTB domain containing 9 [NM_001191462]	Δ*btpB*	2.045	0.0121
zinc finger protein 296 [NM_001192262]	Δ*btpB*	2.538	0.0158
zinc finger protein 30 homolog [XR_083922]	Δ*btpB*	2.295	0.0294
LB02718.CR_O08 GC_BGC-27 cDNA clone IMAGE:8313058 [DV891009]	Δ*virB2*	2.998	0.0025
FtsJ methyltransferase domain containing 2 [NM_001082430]	WT	2.043	0.0280
hypothetical LOC618701 [NM_001099723]	WT	2.316	0.0041
lin-28 homolog B [XM_612469]	WT	2.024	0.0152
sterol regulatory element Binding Protein family member (sbp-1)-like [XM_583656]	WT	2.087	0.0268
zinc finger protein 213 [ENSBTAT00000037526]	WT	2.506	0.0012
zinc finger protein 42. transcript variant 7 [XM_872583]	WT	2.302	0.0396
**Unclassified**			
4105740 BARC 9BOV cDNA clone 9BOV30_O11 5′ [CK974693]	Δ*btpB*	2.381	0.0330
4121344 BARC 8BOV cDNA clone 8BOV_34C06 5′ [CN787257]	Δ*btpB*	2.189	0.0149
chromosome 16 open reading frame 7 [XM_592406]	Δ*btpB*	2.099	0.0197
hypothetical protein LOC616908 [NM_001076437]	Δ*btpB*	2.311	0.0208
LRRN4 C-terminal like [NM_001195069]	Δ*btpB*	2.024	0.0208
Q9V5V8_DROME (Q9V5V8) CG13214-PA [TC333751]	Δ*btpB*	2.704	0.0059
1429623 MARC 7BOV cDNA 3′ [DR113203]	Δ*virB2*	2.193	0.0128
4105740 BARC 9BOV cDNA clone 9BOV30_O11 5′ [CK974693]	Δ*virB2*	2.706	0.0170
AL450489 match: proteins: Q8VCL4 Q9CUK0 Q9Y4E5 [TC317712]	Δ*virB2*	2.001	0.0179
family with sequence similarity 3. member D. transcript variant 2 [XM_865037]	Δ*virB2*	2.144	0.0251
hypothetical protein LOC616908 [NM_001076437]	Δ*virB2*	2.558	0.0058
LB01613.CR_H15 GC_BGC-16 cDNA clone IMAGE:8082593 [DT810899]	Δ*virB2*	2.035	0.0297
LB01652.CR_A20 GC_BGC-16 cDNA clone IMAGE:8385118 5′ [EH174578]	Δ*virB2*	2.094	0.0009
misc_RNA (LOC785410). miscRNA [ENSBTAT00000025153]	Δ*virB2*	2.041	0.0060
similar to Myeloid-associated differentiation marker [NM_001101279]	Δ*virB2*	2.177	0.0396
chromosome 10 open reading frame 120 ortholog [NM_001076476]	WT	2.237	0.0331
hypothetical LOC619120 [XM_871440]	WT	2.208	0.0093
hypothetical LOC785309 [XM_001253376]	WT	2.028	0.0256
LB01613.CR_H15 GC_BGC-16 cDNA clone IMAGE:8082593 [DT810899]	WT	2.243	0.0250
MIPOL1 protein [ENSBTAT00000000857]	WT	2.571	0.0281
misc_RNA (LOC524074). miscRNA [ENSBTAT00000000329]	WT	2.040	0.0279
Q9V5V8_DROME (Q9V5V8) CG13214-PA. isoform A [TC333751]	WT	2.541	0.0239

*Functional classification generated by Funcat (http://mips.helmholtz-muenchen.de/genre/proj/mfungd/Search/Catalogs/searchCatfirstFun.html);

**WT = wild type *Brucella abortus* 2308.

These results indicated that downregulation of host trophoblastic cell transcripts at early stages of infection is directly or indirectly associated with the *virB* Type IV secretion system and BtpB.

### Wild type *B. abortus* impairs host transcription of genes related to immune response when compared to *ΔvirB* and *ΔbtpB* mutants

**Figure 4:**
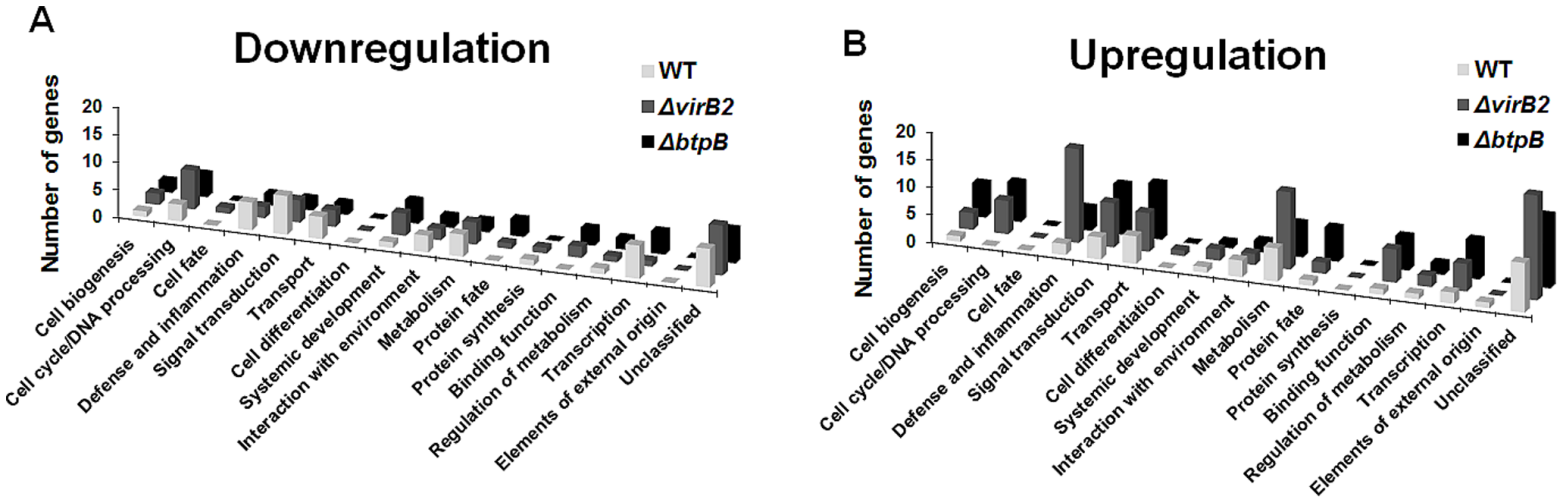
Functional classification of genes with significant changes in transcription levels in bovine trophoblastic cells at 4 hours after infection with wild type, Δ*virB2*, and Δ*btpB B. abortus*. (A) Downregulated genes, and (B) upregulated genes in comparison to mock-infected controls.

Differentially expressed transcripts of trophoblastic cells in response to infection with wild type *B. abortus* or the Δ*virB2* and Δ*btpB* mutant strains were functionally classified (Tables 2 and 3). Explants infected with wild type *B. abortus* had the highest number of downregulated genes that are related to signal transduction (7/43; 16.2%), transcription (6/43; 13.9%), and defense and inflammation (5/43; 11.6%). Upregulated transcripts were related to metabolism (6/37; 16.2%), transport (5/37; 16.2%), and signal transduction (4/37; 10.8%). Explants infected with the Δ*virB2* mutant strain had downregulated transcripts mostly in the following categories: cell cycle/DNA processing (7/44; 15.9%), metabolism (4/44; 9.1%), and signal transduction (4/44; 9.1%), and upregulated genes related to defense and inflammation (17/94; 18.0%), metabolism (14/94; 14.8%), and signal transduction (8/94; 8.5%). Explants infected with the Δ*btpB* strain had larger numbers of downregulated transcripts associated with cell cycle/DNA processing (4/38; 10.5%), transcription (4/38; 10.5%), and systemic development (4/38; 10.5%). Genes upregulated were classified as transport-related (10/79; 12.6%), signal transduction (9/79; 11.3%), and cell cycle/DNA processing (7/79; 8.8%) (Figure 4, Table 3). Interestingly, while in wild type *B. abortus*-infected explants most of the differentially expressed transcripts that were classified as associated with defense and inflammation were downregulated (5/7; 71.4%), explants infected with either the Δ*virB2* or Δ*btpB* strains had mostly upregulated transcripts in that category (17/19; 89.4%), supporting the idea that infection by *B. abortus* 2308 causes a downregulation of the immune response at early stages of infection in order to prevent a robust inflammatory response. Therefore, deletion of the virulence genes *virB2* and *btpB* results in strains that trigger increased transcription of several genes related to the immune response.

**Table 3:** Genes with significant increase in transcription level in bovine trophoblastic cells (chorioallantoic membrane explants) infected with wild type (strain 2308), Δ*virB2*, or Δ*btpB* in comparison to uninfected controls at 4 hours post infection.

Function* and GenBank identification	*B. abortus i*nfection**	Fold Change	P value
**Cell biogenesis**			
actin. gamma 2. smooth muscle. enteric [NM_001013592]	Δ*btpB*	3.388	0.0139
calponin 1. basic. smooth muscle [NM_001046379]	Δ*btpB*	3.047	0.0061
LysM. putative peptidoglycan-binding. domain containing 3 [NM_001192982]	Δ*btpB*	2.404	0.0076
myosin. heavy chain 11. smooth muscle [NM_001102127]	Δ*btpB*	2.986	0.0016
tropomyosin 1 (alpha) [NM_001013590]	Δ*btpB*	2.048	0.0243
tropomyosin 2 (beta) [NM_001010995]	Δ*btpB*	2.710	0.0047
keratin 6A [NM_001083510]	Δ*virB2*	3.474	0.0202
matrilin 3 [XM_591137]	Δ*virB2*	5.058	6 E-05
myosin light chain 2a-like (LOC789339) [XM_001256122]	Δ*virB2*	2.817	0.0170
LysM. putative peptidoglycan-binding. domain containing 3 [NM_001192982]	WT	2.204	0.0357
**Cell cycle/DNA processing**			
esophageal cancer related gene 4 protein [NM_001038113]	Δ*btpB*	3.599	0.0450
establishment of cohesion 1 homolog 2-like (LOC786089 [XM_001253877]	Δ*btpB*	2.120	0.0201
HUMRECQ DNA helicase [TC350379]	Δ*btpB*	2.148	0.0214
NDC80 homolog. kinetochore complex component [XM_582722]	Δ*btpB*	2.182	0.0184
PC4 and SFRS1-interacting protein [ENSBTAT00000036721]	Δ*btpB*	2.024	0.0350
polycomb group ring finger 5 [NM_001077980]	Δ*btpB*	2.910	0.0040
SRY (sex determining region Y)-box 7 [XM_615317]	Δ*btpB*	2.122	0.0205
cAMP responsive element modulator [NM_001034710]	Δ*virB2*	2.059	0.0380
histone cluster 1. H1d (HIST1H1D) [NM_001101066]	Δ*virB2*	2.292	0.0155
nuclear receptor coactivator 7 [ENSBTAT00000049978]	Δ*virB2*	2.345	0.0433
centromere-binding factor 5 like PUA domain containing protein with a type I pseudouridine synthase domain [TC372398]	Δ*virB2*	6.112	0.0379
SAMD9_HUMAN Sterile alpha motif domain-containing protein 9 [TC347659]	Δ*virB2*	2.119	0.0026
T-box 19 mRNA [NM_001075663]	Δ*virB2*	2.135	0.0244
**Defense and inflammation**			
CCAAT/enhancer binding protein (C/EBP). epsilon [NM_001192808]	Δ*btpB*	2.494	0.0084
complement factor H (CFH) [NM_001033936]	Δ*btpB*	3.172	0.0291
tumor necrosis factor (ligand) superfamily. member 10-like [XM_583785]	Δ*btpB*	3.290	0.0267
tumor necrosis factor receptor superfamily. member 13C [NM_001193192]	Δ*btpB*	2.692	0.0317
apolipoprotein L. 3 [NM_001100351]	Δ*virB2*	3.191	0.0168
chemokine (C-C motif) ligand 5 mRNA [NM_175827]	Δ*virB2*	2.121	0.0331
chemokine (C-C motif) receptor-like 2 [NM_001075732]	Δ*virB2*	2.165	0.0295
chemokine (C-X-C motif) receptor 5 [NM_001011675]	Δ*virB2*	2.076	0.0285
chemokine binding protein 2 [NM_001015581]	Δ*virB2*	2.068	0.0105
complement component 3a receptor 1 [NM_001083752]	Δ*virB2*	2.851	0.0072
Fc fragment of IgG binding protein [XM_614095]	Δ*virB2*	2.072	0.0406
fms-related tyrosine kinase 3-like [XM_590263]	Δ*virB2*	2.652	0.0419
heat shock 70 kDa protein 1-like [NM_001167895]	Δ*virB2*	2.150	0.0015
interleukin 1 family. member 6 (epsilon)-like (IL1F6) [XM_601728]	Δ*virB2*	4.025	0.0148
interleukin 15 [NM_174090]	Δ*virB2*	5.250	0.0004
interleukin 2 receptor. alpha [NM_174358]	Δ*virB2*	2.669	0.0156
MPV17 mitochondrial membrane protein-like [NM_001046602]	Δ*virB2*	2.134	0.0018
radical S-adenosyl methionine domain containing 2 [NM_001045941]	Δ*virB2*	2.824	0.0480
rCG28728-like (LOC533818) Protein Gbp4 [XM_001789771]	Δ*virB2*	2.302	0.0076
transmembrane 4 L six family member 19 [ENSBTAT00000057570]	Δ*virB2*	4.074	0.0023
tumor necrosis factor receptor superfamily. member 9 [NM_001035336]	Δ*virB2*	4.246	0.0294
heat shock 70 kDa protein 1-like [NM_001167895]	WT	2.017	0.0055
toll-like receptor 6 [NM_001001159]	WT	2.111	0.0407
**Cell differentiation**			
neuropilin 2 [NM_001193237]	Δ*virB2*	2.681	0.0464
**Signal transduction**			
A kinase (PRKA) anchor protein 12 [XM_591518]	Δ*btpB*	2.152	0.0478
ADAM metallopeptidase with thrombospondin type 1 motif. 6 [NM_001193016]	Δ*btpB*	2.194	0.0198
GRM1 protein Fragment [ENSBTAT00000057021]	Δ*btpB*	2.779	0.0282
olfactory receptor Olr1242-like (LOC788607) [XM_001255616]	Δ*btpB*	2.001	0.0184
olfactory receptor. family 11. subfamily G. member 2-like [XM_002690673]	Δ*btpB*	2.122	0.0021
olfactory receptor. family 2. subfamily A. member 4-like [XM_001790464]	Δ*btpB*	2.813	0.0035
Q2ABB1_BOVIN (Q2ABB1) Bitter taste receptor. partial (12%) [TC320439]	Δ*btpB*	2.066	0.0299
Ras-related associated with diabetes [NM_001045913]	Δ*btpB*	2.394	0.0208
spermatogenesis associated 7 [NM_001098928]	Δ*btpB*	2.411	0.0332
brain and acute leukemia. cytoplasmic [NM_001083508]	Δ*virB2*	2.854	0.0281
cerebellin 3 precursor [NM_001079603]	Δ*virB2*	2.967	0.0233
draxin [NM_001195012]	Δ*virB2*	2.111	0.0437
EDAR-associated death domain [ENSBTAT00000061420]	Δ*virB2*	4.127	0.0499
G protein-coupled receptor 115 [NM_001143875]	Δ*virB2*	3.983	0.0298
GRM1 protein Fragment [ENSBTAT00000057021]	Δ*virB2*	2.180	0.0058
prostaglandin E receptor 2 (subtype EP2) [NM_174588]	Δ*virB2*	2.055	0.0338
thromboxane A2 receptor [NM_001167919]	Δ*virB2*	2.225	0.0365
olfactory receptor. family 5. subfamily I. member 1-like [XM_002693698]	WT	2.388	0.0441
olfactory receptor. family 8. subfamily A. member 1-like [XM_581848]	WT	2.131	0.0329
PIGLHHCGB luteinizing hormone receptor precursor variant B [TC374819]	WT	2.041	0.0479
similar to obscurin. cytoskeletal calmodulin and titin-interacting RhoGEF [NM_001102196]	WT	2.094	0.0228
**Transport**			
BC015727 solute carrier organic anion transporter family member 4A1 [TC352480]	Δ*btpB*	2.283	0.0079
ceruloplasmin-like [XM_002685007]	Δ*btpB*	2.025	0.0133
cytochrome P450. family 4. subfamily V. polypeptide 2 [NM_001034373]	Δ*btpB*	2.185	0.0105
hephaestin-like [XM_587920]	Δ*btpB*	2.225	0.0456
Na+/K+ transporting ATPase interacting 2 [NM_001102345]	Δ*btpB*	2.037	0.0496
phosphatidylinositol binding clathrin assembly protein [NM_001101977]	Δ*btpB*	2.132	0.0410
potassium inwardly-rectifying channel. subfamily J. member 10 [NM_001081601]	Δ*btpB*	2.526	0.0063
RAB GTPase activating protein 1-like [NM_001103263]	Δ*btpB*	3.033	0.0415
reticulon 1 [ENSBTAT00000015218]	Δ*btpB*	2.264	0.0315
ryanodine receptor 3 [XM_590220]	Δ*btpB*	3.020	0.0285
AB051866 RIM long form [TC358572]	Δ*virB2*	2.041	0.0086
ATPase. H+ transporting V0 subunit e2 [NM_001097574]	Δ*virB2*	2.007	0.0134
Na+/K+ transporting ATPase interacting 2 [NM_001102345]	Δ*virB2*	2.137	0.0129
potassium inwardly-rectifying channel. subfamily J. member 13 [NM_001193254]	Δ*virB2*	2.176	0.0023
potassium voltage-gated channel. subfamily H (eag-related) [NM_001191394]	Δ*virB2*	2.429	0.0015
small calcium-binding mitochondrial carrier 1-like [XM_609165]	Δ*virB2*	2.154	0.0417
solute carrier organic anion transporter family [XM_002689417]	Δ*virB2*	2.026	0.0265
ATPase. Ca^++^ transporting. plasma membrane 2 [NM_001191245]	WT	2.251	0.0153
component of oligomeric golgi complex 5 [BC149439]	WT	2.143	0.0081
Na+/K+ transporting ATPase interacting 2 (NKAIN2). mRNA [NM_001102345]	WT	3.087	0.0001
sodium-dependent noradrenaline transporter [ENSBTAT00000046433]	WT	2.188	0.0049
unc-13 homolog C [XM_001249446]	WT	2.520	0.0306
**Systemic development**			
shisa homolog 2 [NM_001101265]	Δ*btpB*	2.197	0.0304
NDRG family member 4 [NM_001075695]	Δ*virB2*	2.054	0.0350
Q69Z70_MOUSE (Q69Z70) MKIAA1910 protein (Fragment) [TC349493]	Δ*virB2*	2.288	0.0273
NDRG family member 4 [NM_001075695]	WT	2.533	0.0276
**Interaction with environment**			
BC026119 contactin 4. isoform c precursor [TC348690]	Δ*btpB*	2.747	0.0276
SCO-spondin homolog [NM_174706]	Δ*btpB*	2.103	0.0497
AF311284 erythroid membrane-associated protein [TC330508]	Δ*virB2*	2.943	0.0056
ectonucleotide pyrophosphatase/phosphodiesterase 2 [NM_001080293]	Δ*virB2*	2.208	0.0225
BC026119 contactin 4. isoform c precursor [TC348690]	WT	2.777	3 E-05
ectonucleotide pyrophosphatase/phosphodiesterase 2 [NM_001080293]	WT	2.193	0.0290
kinesin family member 3B-like. transcript variant 3 [XM_863957]	WT	2.778	0.0242
**Metabolism**			
Acp1 protein-like [XM_868782]	Δ*btpB*	3.137	0.0429
ATPase. Na+/K+ transporting. alpha 2 polypeptide [NM_001081524]	Δ*btpB*	2.419	0.0372
glycerol kinase [NM_001075236]	Δ*btpB*	2.55	0.0022
mitochondrial carnitine palmitoyltransferase 1A [FJ415874]	Δ*btpB*	2.069	0.0432
phospholipid scramblase 4 [NM_001081732]	Δ*btpB*	2.3899958	0.0197
transient receptor potential channel 2 [NM_174477]	Δ*btpB*	2.50079	0.0329
acyl-CoA synthetase short-chain family member 2 [NM_001105339]	Δ*virB2*	2.183	0.0024
acylglycerol kinase [NM_001098969]	Δ*virB2*	2.256	0.0417
ankyrin repeat domain 52 [NM_001192530]	Δ*virB2*	2.319	0.0195
cDNA clone IMAGE:8166104 [BC114144]	Δ*virB2*	3.904	0.0002
cDNA clone IMAGE:8233560 [BC153862]	Δ*virB2*	2.413	0.0455
cytochrome P450. family 2. subfamily J. polypeptide 2-like [XM_587546]	Δ*virB2*	2.419	0.0104
glycine-N-acyltransferase [NM_177513]	Δ*virB2*	2.811	0.0005
LOC781710 protein Fragment [ENSBTAT00000056121]	Δ*virB2*	3.699	0.0107
nicotinamide nucleotide adenylyltransferase 2 [NM_001075486]	Δ*virB2*	2.162	0.0180
PG12B_HUMAN Group XIIB secretory phospholipase A2-like protein precursor [TC318659]	Δ*virB2*	2.030	0.0275
Q6UWU2_HUMAN (Q6UWU2) APKK229 [TC386487]	Δ*virB2*	2.204	0.0345
UDP glucuronosyltransferase 1 family. polypeptide A1 [NM_001105636]	Δ*virB2*	3.970	0.0026
WNK lysine deficient protein kinase 2 [XM_582977]	Δ*virB2*	2.059	0.0433
WSC domain containing 1 [XM_617236]	Δ*virB2*	2.211	0.0212
ankyrin repeat domain 52 [NM_001192530]	WT	2.178	0.0400
glutathione S-transferase. theta 3-like [XM_001256131]	WT	2.345	0.0440
PG12B_HUMAN Group XIIB secretory phospholipase A2-like protein precursor [TC318659]	WT	2.260	0.0337
polo-like kinase 3 [NM_001075153]	WT	2.799	0.0023
transient receptor potential channel 2 [NM_174477]	WT	2.067	0.0262
WSC domain containing 1 [XM_617236]	WT	2.024	0.0180
**Protein fate**			
ADAMTS-like 3 [Source:HGNC Symbol;Acc:14633] [ENSBTAT00000006101]	Δ*btpB*	3.114	0.0331
carboxypeptidase E [NM_173903]	Δ*btpB*	3.077	0.0390
F-box protein 16 [NM_001078119]	Δ*btpB*	2.710	0.0311
ubiquitin specific protease 16-like. transcript variant 2 [XM_866110]	Δ*btpB*	2.180	0.0066
von Hippel-Lindau tumor supressor [NM_001110019]	Δ*btpB*	2.279	0.0439
YOD1 OTU deubiquinating enzyme 1 homolog [NM_001080309]	Δ*btpB*	2.509	0.0374
ADAM metallopeptidase with thrombospondin type 1 motif. 3 [NM_001192797]	Δ*virB2*	2.659	0.0124
heat shock 22 kDa protein 8 [NM_001014955]	Δ*virB2*	2.337	0.0349
misc_RNA (LOC613597). miscRNA [ENSBTAT00000019285]	WT	2.315	0.0422
**Binding function**			
ELAV (embryonic lethal. abnormal vision. Drosophila)-like 4	Δ*btpB*	2.126	0.0388
multiple C2 domains. transmembrane 2 [BC118493]	Δ*btpB*	2.366	0.0429
PHD finger protein 14 [BC148049]	Δ*btpB*	2.471	0.0011
phosphodiesterase 4D interacting protein [ENSBTAT00000061284]	Δ*btpB*	3.121	0.0365
RNA binding motif protein 20 [NM_001192613]	Δ*btpB*	2.526	0.0167
RUN and FYVE domain containing 4 [NM_001102081]	Δ*btpB*	2.320	0.0400
calcineurin-like phosphoesterase domain containing 1 [NM_001031771]	Δ*virB2*	2.020	0.0497
hypothetical LOC505551 [XM_581851]	Δ*virB2*	2.433	0.0181
ribosomal protein SA-like [XR_083612]	Δ*virB2*	2.037	0.0153
RNA binding motif protein 43 [NM_001099168]	Δ*virB2*	2.186	0.0282
TRIM6-TRIM34 readthrough [NM_001046461]	Δ*virB2*	2.223	0.0417
tripartite motif-containing 55. transcript variant 1 [XM_001789975]	Δ*virB2*	4.279	0.0149
hypothetical LOC505551 [XM_581851]	WT	2.456	0.0446
**Regulation of metabolism**			
ArfGAP with coiled-coil. ankyrin repeat and PH domains 2 [NM_001105337]	Δ*btpB*	2.909	0.0049
RPTOR independent companion of MTOR. complex 2 [NM_001144096]	Δ*btpB*	2.147	0.0249
low density lipoprotein receptor-related protein 8. apolipoprotein e receptor [NM_001097565]	Δ*virB2*	2.630	0.0135
regulator of G-protein signaling 16 [NM_174450]	Δ*virB2*	2.047	0.0101
family with sequence similarity 129. member A [NM_001191282]	WT	2.301	0.0063
**Transcription**			
BDP1 protein Fragment [ENSBTAT00000000471]	Δ*btpB*	2.226	0.0048
calmodulin binding transcription activator 1 [NM_001100294]	Δ*btpB*	2.336	0.0111
pseudouridylate synthase 7 homolog (S. cerevisiae)-like	Δ*btpB*	2.052	0.0460
regulation of nuclear pre-mRNA domain-containing protein 2 (RPRD2)	Δ*btpB*	2.139	0.0382
regulatory factor X 7 [NM_001192820]	Δ*btpB*	2.136	0.0104
TBP-associated factor 11 [XM_867002]	Δ*btpB*	2.747	0.0340
zinc finger protein 667 [NM_001102078]	Δ*btpB*	2.560	0.0113
adenosine deaminase. RNA-specific. B2 [NM_001192588]	Δ*virB2*	2.043	0.0150
BDP1 protein Fragment [ENSBTAT00000000471]	Δ*virB2*	2.320	0.0031
calmodulin binding transcription activator 1 [NM_001100294]	Δ*virB2*	2.064	0.0012
EP300 interacting inhibitor of differentiation 3 [NM_001100312]	Δ*virB2*	2.181	0.0017
zinc finger protein 565 [NM_001102216]	Δ*virB2*	2.141	0.0036
POU class 6 homeobox 2 [NM_001102046]	WT	3.300	0.0430
TOX high mobility group box family member 3 [XM_880075]	WT	2.346	0.0435
**Elements of external origin**			
Q52KE6_MOUSE (Q52KE6) Pgbd5 protein [TC306097]	WT	2.120	0.0246
**Unclassified**			
A17601A FFB cDNA clone A1760 3′ [EE903511]	Δ*btpB*	2.243	0.0274
BP250013A20E1 Soares normalized bovine placenta cDNA [BF042192]	Δ*btpB*	3.374	0.0263
chromosome 15 open reading frame 26 ortholog [NM_001075536]	Δ*btpB*	2.145	0.0133
chromosome 9 open reading frame 30 ortholog [NM_001076019]	Δ*btpB*	2.111	0.0187
hCG1653800-like [XM_002695883]	Δ*btpB*	2.853	0.0319
hypothetical protein LOC100125939 [NM_001105502]	Δ*btpB*	2.131	0.0483
hypothetical protein LOC534992 [NM_001079608]	Δ*btpB*	2.290	0.0212
LB029104.CR.1_D17 GC_BGC-29 cDNA clone IMAGE:8491939 [EE363922]	Δ*btpB*	2.740	0.0270
LB03019.CR_A20 GC_BGC-30 cDNA clone IMAGE:8139166 [DV930234]	Δ*btpB*	2.218	0.0201
misc_RNA (LOC784747). miscRNA [ENSBTAT00000002161]	Δ*btpB*	2.416	0.0075
myeloid-associated differentiation marker-like [XM_608387]	Δ*btpB*	2.139	0.0025
Q4SMS2_TETNG (Q4SMS2) Chromosome 8 SCAF14545 [TC378905]	Δ*btpB*	2.117	0.0304
trophoblast Kunitz domain protein 5-like [XR_083836]	Δ*btpB*	3.024	0.0220
001128BAMA005012HT BAMA cDNA [DY090836]	Δ*virB2*	2.889	3 E-05
596479 MARC 6BOV cDNA 3′ [CB423273]	Δ*virB2*	2.106	0.0237
cDNA clone IMAGE:8190996 [BC119997]	Δ*virB2*	2.108	0.0029
chromosome 15 open reading frame 26 ortholog [NM_001075536]	Δ*virB2*	2.043	0.0057
hCG1653800-like [XM_002695883]	Δ*virB2*	2.780	0.0446
Hw_FAT_14_050513_F05 CF-24-HW fat cDNA library cDNA[DV775976]	Δ*virB2*	2.113	0.0218
hypothetical LOC100140997 [XM_001787456]	Δ*virB2*	2.523	0.0305
hypothetical LOC514143 [XM_591946]	Δ*virB2*	2.760	0.0153
hypothetical LOC524181 [XM_002702390]	Δ*virB2*	2.224	0.0180
hypothetical LOC614176 [XM_865557]	Δ*virB2*	2.209	0.0181
LB004140.C21_N19 GC_BGC-04 cDNA clone IMAGE:9059349 3′ [EV693857]	Δ*virB2*	2.007	0.0298
LB02816.CR_B07 GC_BGC-28 cDNA clone IMAGE:8225577 [DV909965]	Δ*virB2*	2.004	0.0107
LB02963.CR_E02 GC_BGC-29 cDNA clone IMAGE:8476204 [EE364260]	Δ*virB2*	2.854	0.0497
misc_RNA (LOC100140452). miscRNA [ENSBTAT00000053419]	Δ*virB2*	2.851	0.0383
P-glycoprotein Fragment [ENSBTAT00000047541]	Δ*virB2*	3.031	0.0386
putative uncharacterized protein FLJ41210 Fragment [ENSBTAT00000010299]	Δ*virB2*	2.177	0.0388
Q61107_MOUSE (Q61107) Purine nucleotide binding protein [TC353960]	Δ*virB2*	2.383	0.0108
similar to LOC129881 protein [XM_868516]	Δ*virB2*	2.988	0.0424
uncharacterized protein ENSP00000334415 homolog [NM_001110092]	Δ*virB2*	2.042	0.0338
001128BAMA005012HT BAMA cDNA [DY090836]	WT	2.148	0.0013
693743 MARC 6BOV cDNA 3′ [CB442901]	WT	2.629	0.0263
cDNA clone IMAGE:8031759 [BC149666]	WT	2.018	0.0354
coiled-coil domain containing 54 [NM_001105490]	WT	2.043	0.0249
hypothetical protein LOC785476 [NM_001099194]	WT	2.152	0.0139
Q4SID5_TETNG (Q4SID5) Chromosome 5 SCAF14581 [TC371777]	WT	2.299	0.0367
Q5JS37_HUMAN (Q5JS37) OTTHUMP00000018294 [TC345240]	WT	2.127	0.0460
uMC-bcl_0B02-014-e08 Day 14 CL from a pregnant animal bcl cDNA 3′ [CV976853]	WT	2.775	0.0030

*Functional classification generated by Funcat (http://mips.helmholtz-muenchen.de/genre/proj/mfungd/Search/Catalogs/searchCatfirstFun.html);

**WT = wild type *Brucella abortus* 2308.

### Transcription of genes related to immune response during the early stages of infection of bovine trophoblasts with wild type, *ΔvirB2* or *ΔbtpB B. abortus* strains

**Figure 5:**
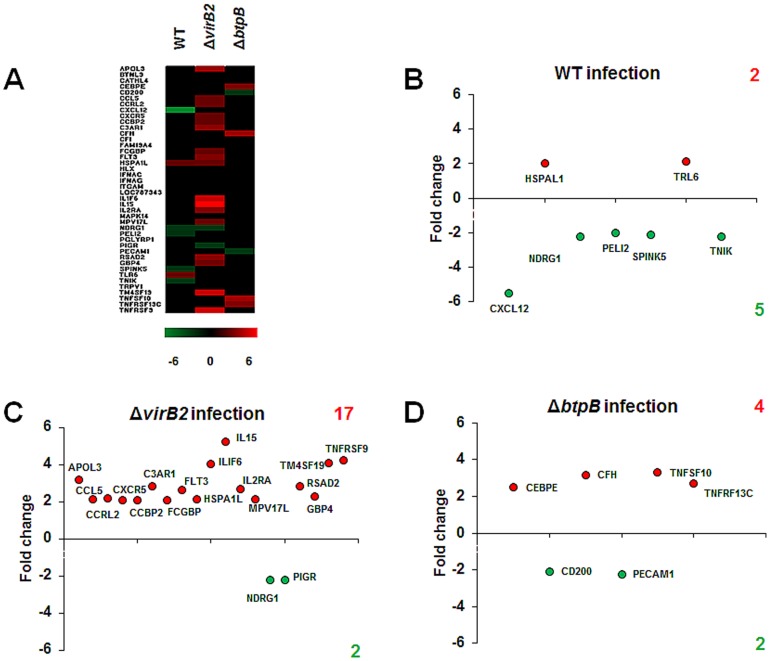
Changes in transcript abundance of defense and inflammation genes during infection of bovine trophoblastic cells with wild type, Δ*virB2* and Δ*btpB B. abortus*, compared to mock-infected controls. (A) Heat map of transcription changes during infections with wild type, Δ*virB2* and Δ*btpB B. abortus*. (B–D) Fold changes in gene transcription of genes classified as defense and inflammation that were significantly (P<0.05) up or downregulated during wild type (B), Δ*virB2* (C), and Δ*btpB* (D) *B. abortus*, compared to mock-infected controls. Fold changes >2 with P<0.05 were considered significant. Abbreviations: apolipoprotein L, 3 (APOL3), butyrophilin-like 9 (BTNL9), cathelicidin 4 (CATHL4), CCAAT/enhancer binding protein (C/EBP), epsilon (CEBPE), CD200 molecule (CD200), chemokine (C-C motif) ligand 5 (CCL5), chemokine (C-C motif) receptor-like 2 (CCRL2), chemokine (C-X-C motif) ligand 12 (CXCL12), chemokine (C-X-C motif) receptor 5 (CXCR5), chemokine binding protein 2 (CCBP2), complement component 3a receptor 1 (C3AR1), complement factor H (CFH), complement factor I (CFI), family with sequence similarity 19 (chemokine (C-C motif)-like), member A4 (FAM19A4), Fc fragment of IgG binding protein (FCGBP), fms-related tyrosine kinase 3-like (FLT3), heat shock 70 kDa protein 1-like (HSPA1L), HLX H2.0-like homeobox (HLX), IFN-alpha C (IFNAC), integrin, alpha M (complement component 3 receptor 3 subunit) (ITGAM), interferon alpha G (IFNAG), interferon-alphaomega-like (LOC787343), interleukin 1 family, member 6 (epsilon)-like (IL1F6), interleukin (IL15), interleukin 2 receptor, alpha (IL2RA), mitogen-activated protein kinase 14 (MAPK14),MPV17 mitochondrial membrane protein-like, nuclear gene encoding mitochondrial protein (MPV17L), N-myc downstream regulated 1 (NDRG1), pellino homolog 2 (PELI2), peptidoglycan recognition protein 1 (PGLYRP1), PIGR polymeric immunoglobulin receptor (PIGR), platelet/endothelial cell adhesion molecule (PECAM1), radical S-adenosyl methionine domain containing 2 (RSAD2), rCG28728-like (GBP4), serine peptidase inhibitor, Kazal type 5 (SPINK5), toll-like receptor 6 (TLR6), TRAF2 and NCK interacting kinase (TNIK), transient receptor potential cation channel subfamily V member 1-like, transcript, variant 2 (TRPV1), transmembrane 4 L six family member 19 (TM4SF19), tumor necrosis factor (ligand) superfamily, member 10-like (TNFSF10), tumor necrosis factor receptor superfamily, member 13C (TNFRSF13C), tumor necrosis factor receptor superfamily, member 9 (TNFRSF9).

Considering that acute inflammation in the placenta is a hallmark of *B. abortus* infection in cattle [7] and that *B. abortus* influences expression of proinflammatory transcripts by bovine trophoblastic cells [13], here we focused the analysis of differentially expressed transcripts associated with defense and inflammation. The microarray data presented above demonstrated downregulation of transcripts in wild type *B. abortus*-infected explants that included chemokines, genes involved in signaling pathways by TLR, in regulation of proliferation and cellular differentiation, cellular response to stress and anti-inflammatory responses. Only two genes in these categories had significantly increased transcription, namely: Toll-like receptor 6 (TLR6) and heat shock 70 kDa protein 1-like (HSPA1L) (Figure 5B). In contrast, trophoblastic cells infected with either the Δ*virB2* mutant strain or the Δ*btpB* strain had a significant increase of transcripts of cytokines and chemokines as well as genes associated with the complement cascade (Figure 5C). Transcription of only two genes related to defense and inflammation were significantly decreased, namely platelet/endothelial cell adhesion molecule (PECAM1) and CD200 molecule (C200) (Figure 5D).

### Validation of the microarray data with qRT-PCR

**Figure 6:**
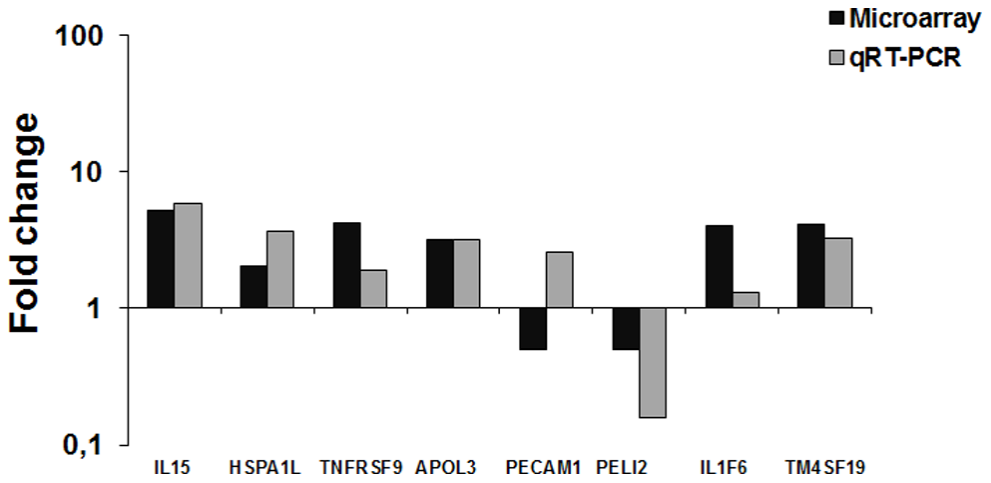
Validation of the microarray results by real-time qRT-PCR. Bars represent the average of fold change values from three independent experiments. Chorioallantoic membranes were infected with wild type *B. abortus* for evaluation of HSPA1L and PELI2 expression; with the Δ*virB2 Brucella abortus* strain for evaluation of IL15, TNFRSF9, IL1F6, and TM4SF19; and with Δ*btpB Brucella abortus* strain for evaluation of PECAM1. Abbreviations: interleukin 15 (IL15), heat shock 70 kDa protein 1-like (HSPA1L), tumor necrosis factor receptor superfamily, member 9 (TNFRSF9), apolipoprotein L, 3 (APOL3), pellino homolog 2 (PELI2), platelet/endothelial cell adhesion molecule (PECAM1), interleukin 1 family, member 6 (epsilon)-like (IL1F6), transmembrane 4 L six family member 19 (TM4SF19).

In order to validate the results obtained with the microarrays analysis, selected differentially expressed transcripts were evaluated by qRT-PCR. Genes encoding IL15, HSPA1L, TNFRSF9, APOL3, PECAM1, PELI2, IL1F6, and TM4SF19 were amplified. Six of the eight selected genes had results that were parallel to those observed by microarray analysis (Figure 6).

## Discussion

This study provided further evidence that *B. abortus* is capable of actively modulating the host innate immune response during the early stages of infection in target cells that are highly relevant for disease transmission, i.e. bovine trophoblasts. A previous study from our group demonstrated that *B. abortus* is able to modulate the innate immune response of bovine trophoblastic cells by suppressing the expression of proinflammatory cytokines and chemokines at early stages of infection [13]. In this study we expanded this notion by demonstrating that the absence of a functional T4SS due to deletion of *virB2* as well as deletion of the *btpB* gene impairs the ability of *B. abortus* to suppress transcription of proinflammatory genes at early stages of infection in bovine trophoblasts. Although suppression of a proinflammatory response by trophoblastic cells may conflict with the fact that *B. abortus* causes acute placentitis in pregnant cows [7], our previous study [13] also demonstrated expression of proinflammatory chemokines at later stages of infection (i.e., 12 h after inoculation) in *B. abortus*-infected CAM explants, with a pattern that is similar to that observed *in vivo* in the placentomes of experimentally infected pregnant cows [13].

BtpB is known to interfere with innate immunity, since it inhibits TLR signaling in dendritic cells [19]. Our results suggest that BtpB could play a similar role in trophoblastic cells, suppressing an innate immune response. The *virB* operon- encoded T4SS is required for *Brucella* spp. to interfere with intracellular trafficking, which mediates exclusion of lysosomal markers from the *Brucella*-containing vacuole, and ultimately allows the pathogen to reach its intracellular replication niche [26]–[28]. Therefore, we hypothesize that the marked differences in host transcriptional profile when trophoblastic cells are infected with a *Brucella* strain lacking a functional T4SS is likely to be a result of differences in intracellular trafficking. Since *virB* mutants are killed and degraded, liberation of TLR ligands from degraded *virB* mutant bacteria couldcontribute to the increased proinflammatory transcriptional profile observed in our study [29]. Alternatively, since the VirB T4SS was recently shown to promote translocation of BtpB into host cells, the increased inflammatory signature in cells infected with the *virB2* mutant could result from reduced translocation of BtpB to the cytosol of infected trophoblasts within the CAM explants, and consequently, reduced suppression of TLR signaling.

Confirming previous results from our group that demonstrated that *B. abortus* inhibits a proinflammatory responses in infected bovine CAM at early stages of infection [13], microarray analysis revealed decreased transcription of genes related to immune response and cellular stress such as chemokine (CXC motif) ligand 12 (CXCL12), Pellino homolog 2 (PELI2), TRAF2 and NCK interacting kinase (TNIK), N-myc downstream regulated 1 (NDRG1) and serine peptidase inhibitor, Kazal type 5 (SPINK5). CXCL12, or stromal-derived factor 1 (SDF-1), is the only ligand for CXCR4 and its decrease may affect various biological processes, including hematopoiesis, cardiogenesis, vascular and neuronal development (processes that may be relevant for fetal development), and traffic of immune cells [30]. In trophoblastic cells infected with *B. abortus*, the reduction of CXCL12 can also cause decreased interaction with CXCR4, which can be deleterious for the developing fetus and affect immune responses. PELI2 is involved in signaling pathways by TLR1, TLR2, TLR4 and IL-1 by interaction with the complex containing IRAK kinases and TRAF6. It mediates polyubiquination of IRAK1 and can activate MAP (mitogen activated protein) kinase pathways. TNIK, in turn, is regulated by TRAF2, and it is induced by stress, external stimuli and by signal transducers like JNK and NF-κB after stimulation by TNF-α. It also acts protecting cells from apoptosis [31]. NDRG1 is involved in regulation of cellular proliferation and differentiation, as well as cellular response to stress. In human trophoblastic cells, this gene promotes cell viability and protection against injury due to hypoxia, a condition that is commonly associated with placental injury and impaired fetal development [32]. SPINK5 is a serine protease inhibitor, probably related to an anti-inflammatory response. In humans, it is important for protection against pathogens, and it plays a role in formation and physiological renewal of the epidermal barrier [33]–[35]. Decreased transcription of these genes confirms the notion of a negative modulation of the immune response at early stages of infection of bovine trophoblastic cells with *B. abortus*. Conversely, two genes within this category had increased transcription in bovine trophoblastic cells infected with *B. abortus*: TLR6 and HSPA1L. Recently, a study showed the importance of TLR6 in triggering the innate immune response against *B. abortus in vivo* and activation of dendritic cells and production of proinflammatory cytokine [36]. HSPA1L (70 kDa heat shock protein 1-like - Hsp70) is a chaperone that may play a role in the internalization of *Brucella* sp. Tropism of *B. abortus* for placental tissues has important implications for the occurrence of *B. abortus*-induced abortion, although the molecular mechanism for this tropism is unknown. Watanabe et al. [37] demonstrated that heat shock cognate protein (Hsc70) plays a role in *Brucella* sp. internalization in trophoblastic cells. The administration of anti-Hsc 70 to pregnant mice prevents abortion [37]. In this study there was increased transcription of this gene in trophoblastic cells infected with the Δ*virB* strain, supporting the notion that Hsp70 may be involved at early stages of *Brucella* infection (4 hpi).

The *virB* operon encodes structural components of the T4SS, and therefore it is required for secretion of effector molecules. The T4SS is required for persistence of *Brucella* spp. *in vivo*, and for intracellular survival in macrophages, which are considered one of the primary target cells for *Brucella* infection. Induction of the T4SS expression occurs after the initial acidification of the *Brucella*-containing vacuole (BCV). Moreover, the absence of markers of phagolysosomes in BCVs as well as the maturation of compartments derived from the endoplasmic reticulum are mediated by the T4SS of *Brucella* [15], [26]–[28]. Thus, the *virB* operon is related to survival and multiplication of *Brucella* in host cells, since *virB* mutant strains fail to multiply and localize to a lysosomal compartment [38], [39]. This study demonstrated that bovine trophoblastic cells infected with the Δ*virB* mutant strain had significant increases in transcription of several proinflammatory genes at early stages of infection, when compared to trophoblastic cells infected with the wild type strain, although the elucidation of the mechanism of this phenotype is beyond the scope of this study, our data support the notion that suppression of proinflammatory responses by trophoblastic cells that is induced by *B. abortus* apparently requires a functional T4SS.

Transcripts of several proinflammatory cytokines and chemokines were significantly increased in trophoblastic cells infected with *B. abortus* Δ*virB* compared to uninfected cells. These transcripts included interleukin 15 (IL15), interleukin 1 family, member 6 (epsilon)-like (IL1F6), interleukin 2 receptor alpha (IL2RA), tumor necrosis factor receptor superfamily, member 9 (TNFRSF9) and chemokines such as chemokine (CC motif) receptor-like 2 (CCRL2), chemokine (CC motif) ligand 5 (CCL5), chemokine binding protein 2 epsilon (CCBP2), chemokine (CXC motif) receptor 5 (CXCR5). CXC chemokines act primarily on neutrophil chemotaxis, whereas CC chemokines are chemoattractants for monocytes, lymphocytes, and eosinophils [40]. There was also an increased transcription of the gene encoding Complement component 3a receptor 1 (C3AR1), which is the 3a peptide receptor, one of the proteins of the complement cascade that opsonizes pathogens, and induces a series of inflammatory responses that help fight infection [41]. Opsonization of *B. abortus* influences the internalization of this pathogen by phagocytic cells [42], [43]. Opsonized bacteria are internalized via receptors for complement and Fc and are more susceptible to the bactericidal action of the macrophages than non-opsonized bacteria which, in turn, are internalized via fibronectin receptor [42], [43]. In the first case, most of the internalized bacteria are destroyed within phagolysosomes before reaching the sites of intracellular multiplication [42], [43]. Therefore, the route of internalization interferes with the intracellular traffic in professional phagocytic cells [43], although opsonized *B. abortus* is capable of surviving intracellularly in human macrophages [44]. Apolipoprotein L, 3 (APOL3) transcripts were also increased in Δ*virB B. abortus* -infected trophoblasts. APOL3 is part of the apolipoproteins family. Increase in APOL proteins is related to signaling by different pro-inflammatory molecules including IFN-α [45], IFN-β [46], IFN-γ [47], and TNF-α [48], which suggests that APOL proteins are involved in immune response.

To a lesser extent when compared to Δ*virB B. abortus* infection, bovine trophoblastic cells infected with Δ*btpB B. abortus* also had increased mRNA levels of genes related to inflammation. When compared to non-infected controls, there was increased transcription of genes related to complement and also of the TNF family. Interestingly, these results contrast with the transcription profile of the spleen from mice infected with *B. abortus* or *B. melitensis* at 3 days post infection, in which there is a T4SS-dependent proinflammatory response [49]. The *btpB* gene is present in *Brucella* and encodes a protein with a TIR-domain, and it inhibits innate immune response probably by binding to MyD88, restricting the TLR signaling and therefore, it contributes to the control of inflammation and establishment of infection [19]. Salcedo et al. [19] reported that a *virB* mutant translocated less of BtpA and BtpB into mouse macrophages. Therefore, the increased expression of inflammation-related transcripts could be related to reduced translocation of the Btp proteins into infected trophoblasts. Furthermore, since *virB* mutants are killed more efficiently after uptake by cells, this could release more microbe associated molecular patterns (MAMPs) into the phagolysosome where they can be detected by TLRs. Two genes belonging to the TNF family had significant increases in transcription: tumor necrosis factor receptor superfamily, member 13C (TNFRSF13C), which is associated with increased survival of B cells *in vitro* and regulating the population of peripheral B cells, and tumor necrosis factor (ligand) superfamily, member 10-like (TNFSF10), is a member of TNF family of cytokines and primarily related to apoptosis. TNFSF10 playing important roles in regulating cell death, immune response, and inflammation. TNFSF10 binding to its receptors promotes activation of MAPK8/JNK, caspase 8, and caspase 3 [50]–[52]. There was also an increased transcription of the complement factor H (CFH), which is a member of the Regulator of Complement Activation (RCA) cluster. It plays an essential role in the regulation of complement activation, restricting the activation of the complement cascade innate immune response against pathogens and CCAAT/enhancer binding protein (C/EBP) epsilon (CEBPE). It is also a critical mediator of myelopoiesis and it is related to the functional maturation of neutrophils and monocytes/macrophages [53]–[55].

In conclusion, our study demonstrated that infection with *B. abortus* induces a downregulation of the bovine trophoblastic innate immune response during the first hours of infection. The *virB*-encoded T4SS, and to a lesser extent the *btpB* gene, play a direct or indirect role in this mechanism.
